# Post-Kidney Transplant Cancer: A Real-World Retrospective Analysis From a Single Italian Center

**DOI:** 10.3389/ti.2024.13220

**Published:** 2024-08-20

**Authors:** Giulia Vanessa Re Sartò, Carlo Alfieri, Laura Cosmai, Emilietta Brigati, Mariarosaria Campise, Anna Regalia, Simona Verdesca, Paolo Molinari, Anna Maria Pisacreta, Marta Pirovano, Luca Nardelli, Maurizio Gallieni, Giuseppe Castellano

**Affiliations:** ^1^ Nephrology and Dialysis Unit, ASST Fatebenefratelli Sacco, Milano, Italy; ^2^ Department of Nephrology, Dialysis and Renal Transplantation, Fondazione IRCCS Ca’ Granda Ospedale Policlinico, Milan, Italy; ^3^ Department of Clinical Sciences and Community Health, University of Milan, Milan, Italy; ^4^ Post-Graduate School of Specialization in Nephrology, University of Milan, Milan, Italy; ^5^ Department of Biomedical and Clinical Sciences, Università di Milano, Milano, Italy

**Keywords:** kidney transplant, post-kidney transplant cancer, immunosuppressive therapy, mTOR inhibitors, induction therapy

## Abstract

We describe the epidemiology of cancer after kidney transplantation (KTx), investigating its risk factors and impact on therapeutic management and survival in KTx recipients (KTRs). The association between modification of immunosuppressive (IS) therapy after cancer and survival outcomes was analyzed. We collected data from 930 KTRs followed for 7 [1–19] years. The majority of KTRs received KTx from a deceased donor (84%). In total, 74% of patients received induction therapy with basiliximab and 26% with ATG. Maintenance therapy included steroids, calcineurin inhibitors, and mycophenolate. Patients with at least one cancer (CA+) amounted to 19%. NMSC was the most common tumor (55%). CA+ were older and had a higher BMI. Vasculitis and ADPKD were more prevalent in CA+. ATG was independently associated with CA+ and was related to earlier cancer development in survival and competing risk analyses (*p* = 0.01 and <0.0001; basiliximab 89 ± 4 vs*.* ATG 40 ± 4 months). After cancer diagnosis, a significant prognostic impact was derived from the shift to mTOR inhibitors compared to a definitive IS drug suspension (*p* = 0.004). Our data confirm the relevance of cancer as a complication in KTRs with ATG as an independent risk factor. An individualized choice of IS to be proposed at the time of KTx is crucial in the prevention of neoplastic risk. Finally, switching to mTORi could represent an important strategy to improve patient survival.

## Graphical Abstract

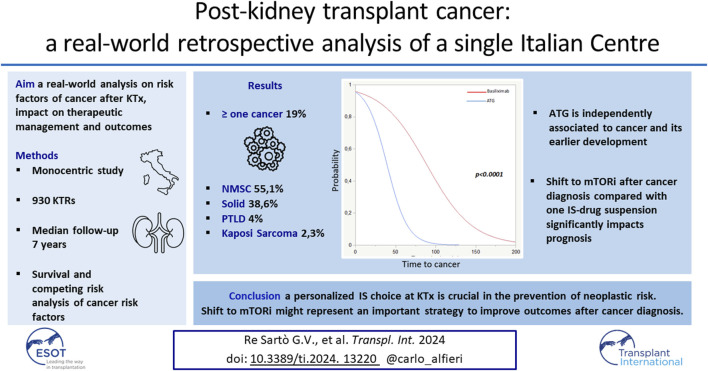

## Introduction

Post-kidney (KTx) transplant cancer is an increasing risk for kidney transplant recipients (KTRs), with incidence rates 2-4 times higher than the general population of similar age and sex [1–3]; this risk escalates with time since transplantation [4]. Malignancies are the second or third leading cause of death in many registries, accounting for up to 56% of deaths in patients with functioning grafts [5]. Recent Italian research identifies cancer as the primary cause of death, accounting for 32.4% of all KTR deaths [6, 7] Variations in mortality suggest differing tumor biology and aggressiveness, and the potential undertreatment of KTRs [8].

Post-KTx malignancy depends on several factors that are related to the patient’s previous individual history and the therapeutic characteristics of the KTx [9]. Cumulative pre-KTx immunosuppressive therapy (IS), advanced age, dialysis vintage, and reduced immunocompetence are relevant risk factors. This setting motivates a challenging evaluation of IS therapy at KTx and individualization of the IS regimen according to patient risk [10]. However, according to the literature, the most appropriate IS management after cancer diagnosis is still lacking in terms of indication quality and evidence level [11].

Our retrospective observational study aimed to describe the epidemiology of post-KTx cancer, investigate its risk factors and impact on management and survival, and provide a real-world long-term analysis from our center.

## Materials and Methods

### Patients and Study Characteristics

We retrospectively analyzed a cohort of 930 kidney transplant recipients at the Fondazione IRCCS Ca’ Granda Ospedale Policlinico Maggiore, Milan, Italy, who underwent transplantation between 2004 and 2021. Patients were followed until September 2023 with a median observation period of 7 [1–19] years. Data were recorded from both paper and electronic hospital records.

### Clinical and Biochemical Evaluations

According to our protocols, blood and urine analyses were performed after a 12-h fast by the nursing staff of the Transplantation Outpatient Clinic.

For each of the 930 KTRs, the following data were recorded:

- at the time of KTx, anthropometric and clinical characteristics: age, sex, type of transplant, dialysis vintage and type, basic nephropathy, presence of diabetes mellitus, body mass index (BMI), steroid use before KTx, and HCV serology.
- at 1 month (T1) and 12 months (T12) after KTx, clinical and biochemical parameters: systolic and diastolic blood pressure, BMI, serum creatinine, hemoglobin, serum albumin, blood glucose, serum uric acid, total cholesterol, HDL cholesterol, triglycerides, 25-OH-vitamin D, and 24-h proteinuria.

Biochemical parameters were measured according to the routine methodology used by our central laboratory. Serum creatinine was assessed by Jaffe’s reaction, whereas urinary protein excretion was assessed by measuring 24-h urine collection protein using the immunoturbidimetric method.

Data on the IS therapy regimen were collected during the first year after KTx; specifically, both the induction IS therapy with basiliximab or ATG and the subsequent maintenance regimen at T1 and T12 with steroids, calcineurin inhibitors (CNI), mycophenolate/mycophenolic acid (MMF), and/or mTORi were fully evaluated. For ATG use, the cumulative dose was expressed in mg/kg; for steroid use, the overall exposure of patients at T1 and T12 was calculated in mg. According to our internal protocol, basiliximab was used in cases of first KTx and low immunologic risk based on donor type and recipient immunologic status. ATG was administered to patients with high immunologic risk or in cases of re-transplantation.

### Cancer Screening and Evaluation

At our center, KTRs undergo regular cancer screening, including an annual abdominal ultrasound and graft examination, along with skin, urological, and gynecological exams. Colorectal cancer screening follows general guidelines. We recorded the time of cancer onset, tumor details, recurrence, staging, management, and therapy. The cohort was divided into CA+ (KTRs who developed cancer) and CA- (KTRs who did not develop cancer). Cancer types included NMSC (graded according to Broder’s system [12]), solid tumors, PTLD, and Kaposi’s sarcoma. We analyzed survival from cancer and KTx, along with patient and graft outcomes post-cancer.

### Principal Outcomes Considered

Patients were followed up for a median time of 7 [1–19] years. At the end of the follow-up, we evaluated as principal outcomes graft loss and death with functioning graft both globally and related to IS modifications after cancer diagnosis.

### Statistical Analysis

Continuous variables were presented as mean and standard deviation or median [25°–75° percentile] for non-normally distributed data. Qualitative data were presented as frequencies and percentages. Group differences were assessed using the Student’s t-test and the Wilcoxon-Mann-Whitney test for normally and non-normally distributed continuous variables, respectively. The nominal variable analysis employed the χ^2^ and Fisher tests. Logistic regression models were used for multivariate analysis of cancer risk factors. Survival analysis utilized Kaplan-Meier curves with the Log-Rank test, a multivariable time-dependent Cox model, and competing risk analysis to assess the mortality risk associated with IS induction therapy. The multivariate analysis variables were determined based on univariate testing. ROC curve analyses with the evaluation of the Youden Index were used for discriminatory analyses. Statistical significance was set at *p* < 0.05. All the statistical analyses in this manuscript were conducted using SPSS 27^®^ software and JMP Pro 15^®^. Treatments and procedures reported herein were in accordance with the ethical standards of the institutional committee where the study was conducted (Fondazione IRCCS Ca’ Granda Ospedale Maggiore Policlinico Ethical Committee, Protocol ID 4759-1837/19), and with the tenets of the Helsinki declaration of 1964 and its later amendments, or comparable ethical standards.

## Results

### Overall Patient Characteristics

The cohort consisted of 930 KTRs, 537 of them men, with a mean age at KTx of 49 ± 13 years. The main clinical characteristics of KTRs are summarized in Table 1.

**TABLE 1:** Clinical characteristics at the time of kidney transplantation in the total cohort.

Clinical variables		Values
Patients - n		930
Sex - n (%)	Male patients	537 (57.7%)
Recipient age at KTx - years		49 ± 13
Transplant donor - n (%)	Deceased	778 (83.6%)
Dialysis type - n (%)	HD PD No	672 (72.2%) 181 (19.4%) 78 (8.4%)
Dialysis vintage - months		52 ± 51
Diabetes pre-KTx - n (%)	Yes No NA	54 (5.8%) 850 (91.3%) 27 (2.9%)
History of HCV infection - n (%)	Yes No NA	37 (3.9%) 565 (61.8%) 328 (35.3%)
Steroids pre-KTx - n (%)	Yes	332 (35.7%)

HD, hemodialysis; PD, peritoneal dialysis; NA, not available.

**FIGURE 1:**
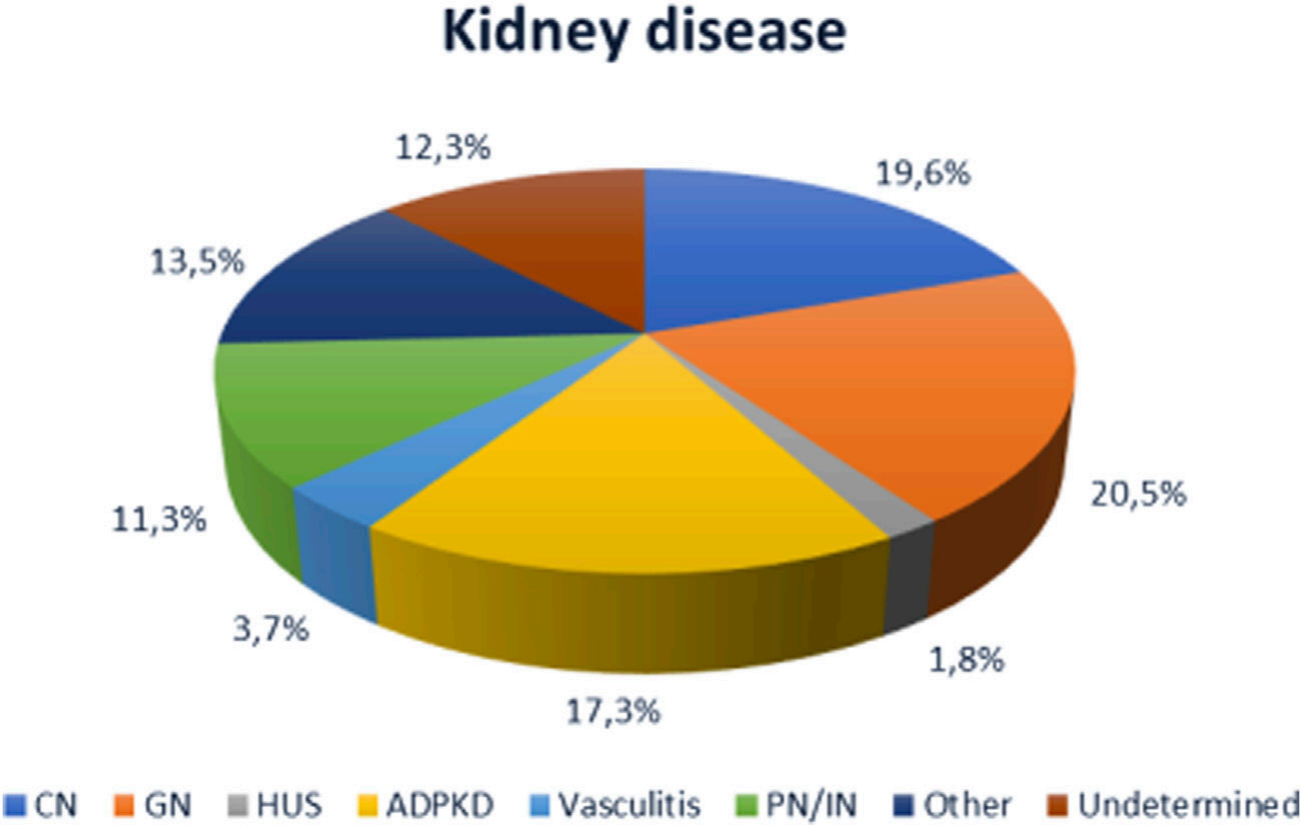
Causes of end-stage renal disease in the cohort (n = 930). CN, chronic nephropathy (diabetic nephropathy, hypertensive renal injury, vasculopathy); GNF, glomerulonephritis; HUS, hemolytic uremic syndrome; ADPDK, autosomal dominant polycystic kidney disease; PN/NI, pyelonephritis and interstitial nephritis.

The majority of patients were on dialysis prior to KTx, with a higher prevalence of hemodialysis and a mean dialysis vintage of 52 ± 51 months. The majority of patients received KTx from a deceased donor. The most common cause of kidney disease in the overall cohort was glomerulonephritis (20.5%) (Figure 1).

Data regarding induction and maintenance IS are shown in Supplementary Table S1. The majority of patients who received induction therapy had been treated with basiliximab (74%) while ATG was administered in 26% of cases, with the latter having a mean individual dose of 4.8 ± 1.1 mg. Of note, between 2004 and December 2015 (KTRs, n = 576), only 10.4% (KTRs, n = 60) of patients received ATG, whereas between 2016 and 2021 (KTRs, n = 355), ATG and basiliximab were used in 53.8% (KTRs, n = 191) and 46.2% of cases, respectively. Maintenance therapy included, in the majority of cases, the use of steroids with a cumulative dose of 1094 ± 438 mg at T1 and 2915 ± 1004 mg at T12; CNI (tacrolimus 91.9%, ciclosporin 7.7%) and mycophenolate/mycophenolic acid (94.6%) were the most represented categories. Only 2.6% of patients were prescribed mTORi. No significant differences were observed in the distribution of IS regimens from T1 to T12.

### Biochemical and Anthropometric Parameters of the Entire Cohort

The biochemical and anthropometric parameters of the entire cohort at T1 and T12 are summarized in Table 2. Among the anthropometric characteristics, BMI at T1 showed a substantial increase during the first year post-KTx. No differences were found in blood pressure control. Kidney function did not differ significantly between T1 and T12. Regarding the biochemical data, the levels of hemoglobin, albumin, uric acid, and 25-OH-vitamin D increased during the 12 months; lipid control remained stable during the first year after KTx.

**TABLE 2:** Anthropometric and biochemical characteristics of the total cohort at T1 and T12.

Variables	T1	T12
Patients – n = 930		
BMI - kg/m^2^	23 ± 3	24 ± 3
SBP - mmHg	130 ± 16	131 ± 17
DBP - mmHg	80 ± 10	80 ± 10
Creatinine - mg/dL	1.5 ± 0.63	1.4 ± 0.46
Proteinuria - g/24 h	0.2 [0.14–0.3]	0.17 [0.1–0.25]
Hb - g/dL	10.9 ± 1.35	12.7 ± 1.6
Albumin - g/dL	4.1 ± 0.42	4.4 ± 0.45
Glucose - mg/dL	89 ± 24	89 ± 24
Uric acid - mg/dL	5.8 ± 1.6	6.6 ± 2.7
Total cholesterol - mg/dL	211 ± 50	195 ± 43
HDL cholesterol - mg/dL	61 ± 19	56 ± 17
Triglycerides - mg/dL	165 ± 84	160 ± 83
25-OH-vitamin D - ng/mL	14.8 ± 8.2	19.9 ± 12.1

BMI, body mass index; SBP, systolic blood pressure; DBP, diastolic blood pressure; Hb, hemoglobin.

### Post-Kidney Transplant Cancer

During the follow-up, 177 KTRs (19%) were diagnosed with at least one cancer, with a mean time to development from KTx of 83 ± 48 months.

Available data showed the occurrence of *de novo* cancer in 113 patients and the recurrence of a pre-KTx tumor in 9 cases; in 55 subjects, cancer could not be defined as *de novo* nor recurrent. NMSC was the most common cancer type (55.1%), while solid tumors were observed in 69 patients (38.6%); PTLD and Kaposi’s sarcoma were diagnosed in 4% and 2.3% of cases, respectively (Supplementary Table S2).

In one-third of the KTRs (n = 58), a second cancer further developed (specifically 36 NMSC, 19 solid tumors, 2 PTLD and 1 Kaposi’s sarcoma), with recurrence in half of the cases (50% *de novo* cancer). The mean time to the onset of the second neoplasia after KTx was 122 ± 53 months. For both first and second tumors, the most prevalent grade of NMSC was G2; among solid cancers, the most frequent primary cancer sites were breast (13.2%), prostate (11.7%), cervix (10.3%), lung (10.3%), and urothelial carcinoma (8.8%). Considering first malignancies, 6% of cases were diagnosed with lymph node involvement and 4% with metastatic disease at diagnosis.

### Comparison Between CA+ and CA-: Clinical and Biochemical Risk Factors for Cancer

Table 3 and Supplementary Table S3 summarize the clinical, biochemical, and anthropometric differences observed between CA+ and CA- during follow-up.

**TABLE 3:** Clinical differences from the comparison of KTRs who developed cancer (CA+) and KTRs without cancer diagnosis (CA-).

Clinical variables		CA- (n = 753)	CA+ (n = 177)	*p*-value
Sex - n (%)	Male patients Female patients	426 (79.3%) 327 (83.2%)	111 (20.7%) 66 (16.8%)	0.08
Transplant donor - n (%)	Deceased Living	620 (79.9%) 131 (86.2%)	156 (20.1%) 21 (13.8%)	**0.04**
Age at KTx - years		47.5 ± 13	54.6 ± 10.6	**<0.001**
Dialysis type - n (%)	HD PD No	522 (80.6%) 139 (76.8%) 68 (87.2%)	125 (19.4%) 42 (23.2%) 10 (12.8%)	0.17
Dialysis vintage - months		51.2 ± 49	57.8 ± 57.1	0.06
Diabetes pre-KTx - n (%)	Yes No	44 (81.5%) 684 (80.5%)	10 (18.5%) 166 (19.5%)	0.51
History of HCV infection - n (%)	Yes No	26 (70.3%) 422 (74.7%)	11 (29.7%) 143 (25.3%)	0.33
Steroids pre-KTx - n (%)	Yes No	269 (81%) 417 (80.8%)	63 (19%) 99 (19.2%)	0.50
Causes of ESRD – n (%)	CN GN HUS ADPKD Vasculitis PN/NI Other Undetermined	145 (79.7%) 154 (80.6%) 16 (94.1%) 118 (73.3%) 23 (67.6%) 91 (86.7%) 107 (84.9%) 99 (86.8%)	37 (20.3%) 37 (19.4%) 1 (5.9%) 43 (26.7%) 11 (32.4%) 14 (13.3%) 19 (15.1%) 15 (13.2%)	**0.011**

HD, hemodialysis; PD, peritoneal dialysis; CN, chronic nephropathy; GN, glomerulonephritis; HUS, hemolytic uremic syndrome; ADPDK, autosomal dominant polycystic kidney disease; PN/NI, pyelonephritis and interstitial nephritis.

Bold value indicates the statistic significance (*p* < 0.05).

CA+ exhibited a statistically significant difference in age, being older than CA- (*p* < 0.001). The incidence of malignancy was higher in men, with a mean age at diagnosis of 54 ± 10 years. Although not of causal meaning, CA+ correlated significantly with KTx from a deceased donor (*p* = 0.04) and dialysis treatment by any method (*p* = 0.06) with a mean dialysis vintage of 57.8 ± 57.1 months. There was no statistically significant difference between the two groups regarding diabetes, steroids pre-KTx or HCV positivity before KTx.

CA+ showed a higher prevalence of vasculitis and ADPKD, as well as basal nephropathy.

Moreover, CA+ correlated with significantly higher BMI values at T1 and T12 (*p* = 0.018 and *p* = 0.08, respectively). No other anthropometric differences were found.

When comparing biochemical variables, no significant differences were found between CA+ and CA- for renal function values such as serum creatinine and proteinuria at T1 and T12. In addition, no significant difference was found for the values of hemoglobin, albumin, blood glucose and uric acid at T1 and T12. However, during the regular management of KTRs, higher levels of total cholesterol at T12 (*p* = 0.034) and lower levels of 25-OH-vitamin D at T1 and T12 (*p* = 0.08 and *p* = 0.033) were reported in CA+ compared to CA-.

### Comparison Between CA+ and CA-: Immunosuppressive Regimen as a Risk Factor for Cancer

In the first year after KTx (Table 4), a significant correlation with higher cumulative therapy with steroids at T1 was found in CA- (*p* = 0.03), a result that was not confirmed at T12. Furthermore, the significance of the results of the IS regimen containing CNI at T1 and T12 remains difficult to interpret considering the almost ubiquitous use of tacrolimus in the maintenance regimens of the entire cohort.

**TABLE 4:** Differences in induction and maintenance of immunosuppressive therapy from the comparison at T1 and T12 of KTRs who developed cancer (CA+) and KTRs without cancer diagnosis (CA-).

Drugs		CA- (n = 753)	CA+ (n = 177)
Induction of immunosuppression			
Basiliximab - % ATG - % ATG dose - mg/kg		522 (56.3%) 186 (20%) 4.92 ± 1.1	142 (15.3%) 29 (3.1%) 4.44 ± 1.2
2016–2021 interval Basiliximab - % ATG - % ATG dose - mg/kg		148 (41.7%) 155 (43.6%) 4.8 ± 0.7	8 (2.2%) 17 (4.7%) 4.66 ± 0.7
Maintenance of immunosuppression			
Ciclosporin - % Tacrolimus - % No - %	T1	41 (4.8%) 635 (74.4%) 1 (0.1%)	24 (2.8%) 151 (17.7%) 2 (0.2%)
Ciclosporin - % Tacrolimus - % No - %	T12	46 (5.7%) 579 (71.8%) 9 (1.1%)	19 (2.4%) 145 (18%) 8 (1%)
MMF-MPA - %	T1 T12	645 (75.5%) 579 (71.8%)	164 (19.2%) 156 (19.4%)
mTORi - %	T1 T12	17 (2%) 34 (4.2%)	5 (0.6%) 13 (1.6%)
Cumulative steroid dose - mg	T1 T12	1109 ± 422 2941 ± 983	1040 ± 492 2817 ± 1076

ATG, antithymocyte polyclonal immunoglobulins; MMF-MPA, mycophenolate-mycophenolic acid; mTORi, inhibitors of the mTOR system.

Regarding induction IS therapy, in the entire follow-up cohort, basiliximab showed a significant correlation with cancer development, a result that was not confirmed when the induction therapy was evaluated between 2016 and 2021. In this specific period, characterized by a homogeneous and comparable administration of two different drugs in induction, the use of ATG proved to be significantly associated with cancer development after KTx.

We did not detect a significant difference in the average individual dose of ATG between the groups, which, according to the Center’s strategy, we attempted to maintain as a target of <5 mg/kg.

### ATG as an Independent Cancer Risk Factor: Multivariate Survival Analysis

In the multivariate analysis, BMI at T1, cumulative exposure to steroid therapy at T12, and use of basiliximab or ATG were included as dependent variables, with post-KTx cancer as the independent variable.

Induction therapy with ATG emerged as an independent and modifiable risk factor with a statistically significant association with cancer development after KTx (*p* = 0.014, HR 3.43, CI 1.287–9.153) (Table 5).

**TABLE 5:** Multivariate analysis using logistic regression models. Independent variable: development of cancer after KTx.

Variables	Regression coefficient	Std error	Hazard ratio	Confidence interval	*p*-value
Recipient age	0.061	0.009	1.063	1.045–1.081	<0.001
BMI T1	0.005	0.026	1.005	0.956–1.056	0.851
Steroids T12	0	0	1.0	1.0–1.0	0.690
ATG	1.233	0.5	3.432	1.287–9.153	**0.014**
Basiliximab	0.313	0.505	1.368	0.508–3.683	0.535

Bold value indicates the statistic significance (*p* < 0.05).

**FIGURE 2:**
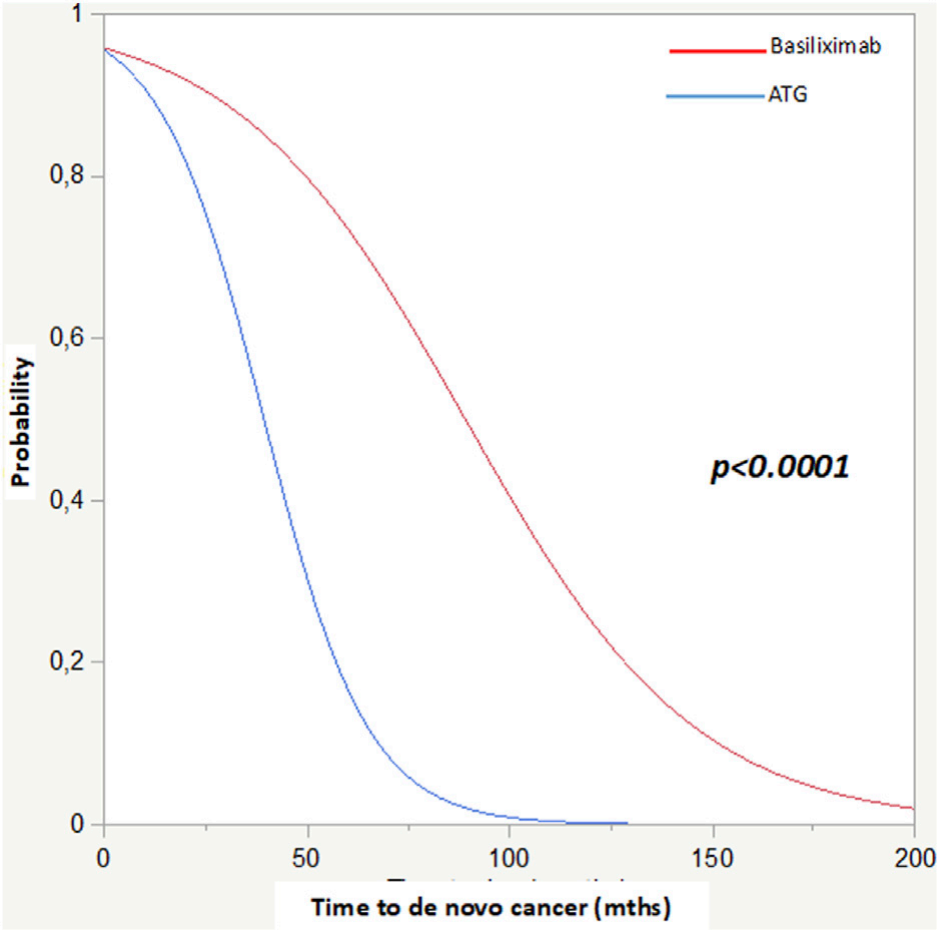
Competing risk analysis: Basiliximab and ATG in relation to time to cancer diagnosis post-KTx.

The critical role of ATG in oncological risk was confirmed by survival analyses. This was first demonstrated in the Kaplan-Meier analysis (*p* < 0.0001) and subsequently confirmed by both the Cox (*p* < 0.01 HR 3.4) and competing risk (*p* < 0.0001, time to cancer onset: basiliximab 89 ± 4 vs*.* ATG 40 ± 4 months) analyses (Figure 2; Table 6).

**TABLE 6:** Cox analysis.

Variables	Regression coefficient	Std error	Hazard ratio	Confidence interval	*p*-value
BMI T1	0.019	0.027	1.019	0.967–1.074	0.480
Steroids T12	0	0	1.0	1.0–1.0	0.981
ATG	1.437	0.556	4.208	1.414–12.521	**0.010**
Basiliximab	−0.207	0.552	0.813	0.276–2.397	0.707

Bold value indicates the statistic significance (*p* < 0.05).

### Therapeutic Strategies for Cancer After KTx: Oncological Treatment

**FIGURE 3:**
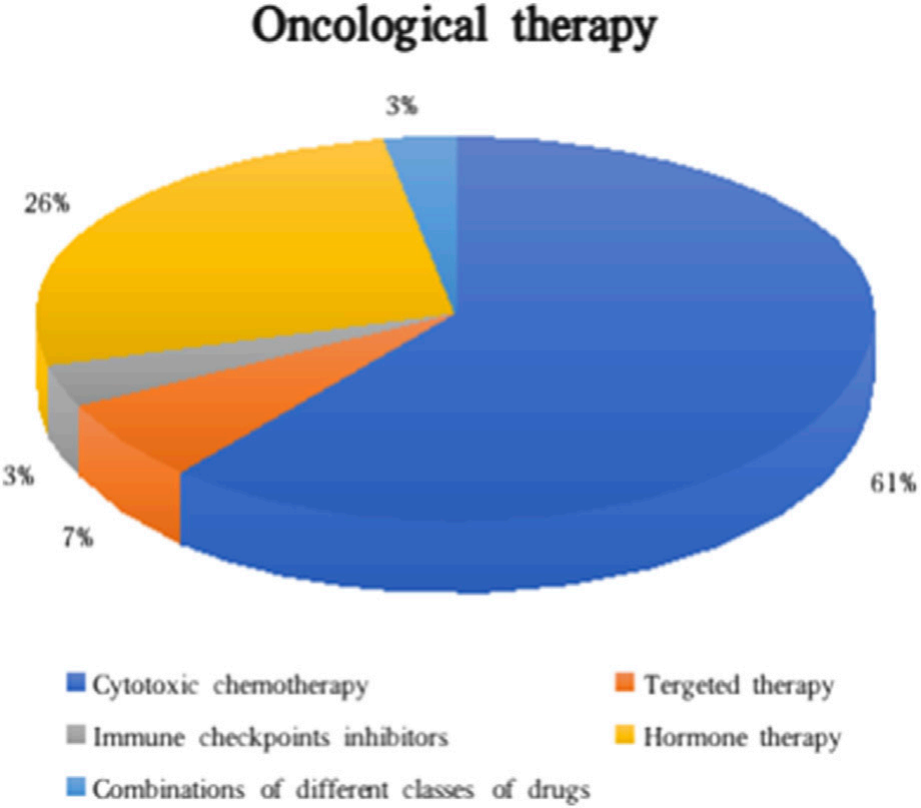
Classes of oncological therapies administered in CA+.

Oncological management for the different types of cancer included a surgical approach (75.1%), radiotherapy (8.4%), and/or active oncological treatment (17.5%). In the majority of individuals who underwent active systemic treatment, cytotoxic chemotherapy (61.3%) was the leading indication, while some CA+ patients started targeted therapy, immunotherapy or a combination of different classes of drugs (Figure 3). In the subgroup with second post-KTx neoplasia, active oncological treatment was initiated in 7 cases. Of note, at least 10 patients did not receive the optimal oncological treatment because of the potential risk of toxicity for the transplanted graft (i.e., no treatment or choice of less nephrotoxic drugs).

At cancer diagnosis, the mean serum creatinine was 1.53 ± 0.74 mg/dL. There were no reports of nephrotoxicity or renal adverse events directly associated with oncological therapy (acute kidney injury, proteinuria, electrolyte imbalance, microangiopathy, etc.); in two cases, acute renal failure of pre-renal origin occurred.

### Therapeutic Strategies in Cancer After KTx: Immunosuppressive Treatment

In 113 cases (65.7%), no major changes in the IS maintenance therapy regimen were made after cancer diagnosis, except for a general reduction in trough levels (trough levels: tacrolimus 3–5 μg/L–cyclosporine 80 μg/L). In total, 39.3% of CA+ subjects were subjected to modifications of the IS therapy, 12.2% of them in combination with the start of an active oncological therapy. These modifications consisted of the introduction in 38 cases (64.4%) of the mTORi regimen, which allowed the serum levels of CNI to be consensually minimized. In 16 cases (27.1%) an antimetabolite drug was permanently discontinued to achieve a dual IS regimen; of the available data, five patients (8.4%) underwent both therapeutic indications.

### Patient Clinical Outcomes

A total of 125 patients (13.4%) experienced graft failure and returned to dialysis after a median time from KTx of 57.3 [8.4–103.1] months. Of these, 9.6% were CA+ and in four cases a cancer-related cause was associated with the return to dialysis (in most cases due to explantation for cancer involving the graft).

During follow-up, 124 patients (13.3%) died, of whom 31.6% were CA+ and 9% CA-. Mortality was mainly due to septic shock (24.1%), cancer-related causes (23.3%), or cardiovascular events (18.5%). Median survival from cancer diagnosis to death was 23 [7.9–59.4] months, with a higher prevalence of cancer-related causes of mortality (51.7%) than all other causes in CA+.

Comparing CA+ and CA-, the median survival from KTx to the end of the follow-up was 148 [88–191] months in CA+ and 82 [45–147] months in CA-, confirming that tumor development and cancer-related mortality are common complications in long-term transplantation. Using competing risk analyses, we investigated the possible relationship between induction therapy and mortality. Interestingly, we found a significantly lower survival in the ATG-treated group (148 ± 2 vs*.* 89 ± 3 months, *p* < 0.0001).

We investigated the correlation between IS therapy strategies following cancer diagnosis and survival outcomes in CA+. Although no significant differences were observed when comparing various modifications in IS therapy, with no change in median survival (60.3 ± 47 vs. 63.3 ± 43 months, *p* = 0.339), a significant prognostic impact was identified regarding changes in the type of IS therapy regimen. Specifically, switching to mTORi and reducing CNI were associated with prolonged overall survival post-cancer diagnosis compared to patients who definitively discontinued one drug, while maintaining a dual therapy approach (67.4 ± 40 vs. 34.4 ± 40 months, *p* = 0.004). This was also investigated, by non-parametric analyses taking into account the type of cancer, dividing our cohort into NMSC and other cancers. Both in the NMSC group and the other cancers group, switching to mTORi and reducing CNI were associated with prolonged post-cancer overall survival: NMSC: 61 ± 35 vs. 34 ± 48 months, *p* = 0.002; other cancers: 61 ± 35 vs. 34 ± 40 months, *p* = 0.01.

No differences in principal anamnestic and biochemical parameters were found between those patients who switched to mTORi and those who did not.

## Discussion

This study aimed to perform a real-world analysis of post-KTx cancer at our transplant Center. The development of at least one cancer occurred in 19% of the KTRs. In an Italian multicenter study with a 25-year follow-up [13], an incidence rate of post-KTx cancer of approximately 13% was reported. In a single-center experience, Gioco et al. reported a cancer incidence of 7.2% with a 7.8 years follow-up [14]. In a recent paper by Srisuwarn et al, an incidence of 6.2% post-transplant cancer was reported [15]. Our results are in line with the trend and the standardized incidence rates of international registries which vary between 1.5 and 3.8 for any post-KTx tumor [1, 3, 6].

According to data reported in the literature, NMSC was the most frequent cancer [11]. The incidence of Kaposi’s sarcoma and PTLD was comparable to other KTR cohorts [16, 17].

A second cancer occurred in 32.7% of CA+ patients at 122 ± 53 months after KTx. Two consecutive NMSCs accounted for more than half of the cases and the majority of recurrences. This suggests a reevaluation of IS maintenance therapy after NMSC, considering the positive impact of switching to mTORi on reducing the first recurrence of squamous cell carcinoma [18, 19].

Age at KTx was a major risk factor. Older age at KTx is associated with a higher absolute risk of cancer because of immune system senescence with reduced immunosurveillance and the greater need for IS in marginal donors [20]. Dialysis vintage, although longer compared to CA-, was not significant (*p* = 0.06) for CA+. However, an increasing dialysis vintage was identified by Wong et al. as a significant risk factor for post-KTx solid tumors with a linear relationship [10].

Among kidney diseases, a greater presence of vasculitis was observed in CA+ subjects. This association, with a limited description in the literature, could be a consequence of the cumulative potency of prior IS treatment added to IS for KTx [21, 22]. In a paper recently published by our group, we observed a significant incidence of post-transplant cancer in vasculitis, which may be due to decreased immunosurveillance caused by a stronger immunosuppressive therapy administered before and after the transplantation [23]. In addition, we demonstrated a correlation between ADPKD and CA+, confirming some findings reported in the literature [24, 25].

A higher BMI at T1 appeared to be statistically significant; one observational study reported higher exposure to tacrolimus in obese KTRs with a substantial dose reduction required at 3 months [26]. Thus, the risk of overexposure to IS therapy may contribute to the increased risk of cancer in KTRs with a high BMI. In any case, since obesity may be independently linked to cancer development [27, 28], we explored this relationship further. We divided our cohort according to the median BMI T1 (23.6 kg/m^2^). No statistical differences were found between those patients above or below the median BMI T1 in terms of time to *de novo* cancer (81 ± 49 vs. 83 ± 46 months, *p* = 0.87) and survival from *de novo* cancer (64 ± 41 vs. 61 ± 46 months, *p* = 0.32). BMI T1 also showed no influence on the type of cancer developed by the patients (*p* = 0.25). We also examined the discriminatory power of BMI T1. The ROC curve analysis, showed a significant ability to identify patients at high risk of developing *de novo* cancer (*p* = 0.003- BMI T1 23.67 Youden index 0.103, with an AUC of 0.56).

Our study shows that ATG significantly affects the risk of cancer post-KTx compared to Basiliximab. ATG use is linked to an increased risk of NMSC, kidney, and lung cancer. [29]; in other studies, ATG use was associated with an increased risk of PTLD [30, 31]. The possible reason for these findings is related to the ability of ATG to accelerate immunosenescence and the decline of cell-mediated immune responses [32]. Careful, individualized choice of induction therapy is essential to mitigate cancer risk, especially in younger recipients, those with a neoplastic history, and considering the immunologic risk profile of both recipient and donor.

We found a high mortality rate among CA+ individuals, with cancer being the leading cause of death in this group and the second leading cause in the entire cohort. Among deceased CA+ individuals, 23% had advanced or metastatic cancer at diagnosis, leading to death within 2 years. These findings highlight cancer as a major cause of death in kidney transplant recipients. The variance in median survival post-transplantation supports international registry observations of increasing long-term cancer-related mortality, which represents a cumulative risk [33]. International registries have already established that cancer represents the second or third cause of death in KTRs, a consequence of better management of infections and cardiovascular disease in the last decades [34, 35]. Our study confirms the negative impact of post-KTx cancer on outcomes and emphasizes the importance of proper screening and surveillance to enable early cancer diagnosis and more effective treatment.

In recent decades, cancer treatments have significantly improved survival and outcomes. However, KTRs are often excluded from pharmacokinetic studies and oncology trials, complicating their treatment. Nonetheless, new drug opportunities should not be dismissed. We observed active oncological treatment in only 17.5% of CA+ cases, mostly with cytotoxic chemotherapy. Contrary to reports in the literature, our study did not record any renal adverse events or rejections due to oncological therapy [36, 37]. The monocentric and retrospective nature of the study limited our ability to gather complete information on the oncological appropriateness of indications, the feasibility of optimal oncological treatment, and data to evaluate outcomes in KTRs. We hypothesized that the lack of precise indications in KTRs and limited nephrology expertise led to a conservative approach. Rousseau et al. described that, according to current guidelines, 11% of cases indicated that KTx and IS interfere with optimal specific cancer treatment [38].

According to our data, a significant improvement in survival, in both the NMSC group and other cancers was observed with the switch to mTORi compared with the second group, although the therapeutic impact of each cancer treatment could not be separated. The indication of mTORi remains controversial; some data recommend mTORi to reduce the risk of the first recurrence of NMSC [39]. In solid malignancies, highly variable data are available [40]; however, one small study found no significant difference in the mTORi group for cancer recurrence, patient survival, and graft function [41]. A meta-analysis of 56 studies reported a positive association between sirolimus and a 40% reduction in cancer risk, particularly a 56% reduction in NMSC risk [42]; however, an unexpected correlation between sirolimus and an increased risk of mortality was described [43]. To date, the introduction of mTORi along with optimal anticancer treatment are the two factors that appear to markedly improve CA+ survival. Further studies will be crucial to identify high-risk cancer patients who will benefit from the use of mTORi.

While our study has a large sample size and extended follow-up in its monocentric design, limitations exist. Nonetheless, our findings could inspire broader multicenter studies to aid in KTx-patient cancer management. Notably, initiating mTORi after cancer diagnosis correlated with improved patient survival. However, the size of our cohort limits us to basic univariate analyses, precluding in-depth examination across various cancer types. Future multicenter investigations may delve into this area of interest.

Our analysis excluded episodes of rejection: no one received ATG therapy for rejection. The retrospective nature of this study limited comprehensive data collection on post-KTx cancer management, including treatment duration, dose adjustments, therapeutic indications based on graft function, and potential no-treatment. Consequently, we could not evaluate the impact of oncological treatment on long-term clinical outcomes in KTRs.

## Conclusion

Post-KTx cancer is a significant complication with notable mortality. Although primarily descriptive, our monocentric study includes a large number of patients. Administering ATG significantly influences early cancer development post-KTx and must be an individualized choice, taking into account recipient and donor characteristics and previous cancer history. If cancer develops, variations in IS therapy and initiations of mTORi should be considered for better outcomes.

## Data Availability

The raw data supporting the conclusions of this article will be made available by the authors, without undue reservation.

## Abbreviations

ADPKD, Autosomal dominant polycystic kidney disease; BMI, Body mass index; CA-, Patients with no cancer diagnosis; CA+, Patients with at least one cancer; CNI, Calcineurin inhibitors; IS, Immunosuppressive therapy; KTRs, Kidney transplant recipients; KTx, Kidney transplant; MMF, Mycophenolate/mycophenolic acid; mTORi, mTOR inhibitors; NMSC, Non-melanoma skin cancer; PTLD, Post-transplant lymphoproliferative disorder.
