# Supplementary material for: Post-Kidney Transplant Cancer: A Real-World Retrospective Analysis From a Single Italian Center
Source: Transpl Int. 2024 Aug 20;37:13220. doi: 10.3389/ti.2024.13220 (PMC11368674; doi:10.3389/ti.2024.13220)

**Supplementary material**

Table S1: Characteristics of immunosuppressive therapy at T1 and T12 in the total cohort.

| **Drugs** | **T1** | **T12** |
| --- | --- | --- |
| **Induction immunosuppression** | | |
| Basiliximab - %  ATG - %  ATG dose - mg/kg | 74%  26%  4,86 ± 1,12 | NA  NA  NA |
| **Maintenance immunosuppression** | | |
| Ciclosporin - %  Tacrolimus - %  MMF-MPA - %  mTORi - % | 7,7%  91,9%  94,6%  2,6% | 8,1%  89,8%  91,2%  5,8% |
| Steroid cumulative dose - mg | 1094 ± 438 | 2915 ± 1004 |

^ATG: polyclonal anti-thymocyte immunoglobulin; MMF-MPA: mycophenolate-mycophenolic acid; mTORi: inhibitors of the mTOR system.^

Table S2: Categorization of post-kidney transplant cancer found in the studied cohort; NMSC: non-melanoma skin cancer; PTLD: post-transplant lymphoproliferative disease.

| **Type of cancer** |  | **Cases n (%)** | **Global %** |
| --- | --- | --- | --- |
| Patients - n |  | 177 |  |
| NMSC | Squamous cell  Basal cell | 27,8%  27,3% | 55,1% |
| Solid tumors | Anus  Bladder  Breast  Cervix uteri  Colon  Kidney  Kidney graft  Larynx  Liver  Lung  Melanoma  Mesothelioma  Pancreas  Prostate  Sarcoma  Testis  Thyroid  Uterus | 1,1%  3,4%  5,1%  4%  2,8%  2,8%  1,1%  0,6%  0,6%  4%  3,4%  0,6%  1,7%  4,5%  1,1%  0,6%  0,6%  1,1% | 38,6% |
| PTLD | Leukemia  Lymphoma  Lymphoma of graft  Multiple myeloma | 1,1%  1,7%  0,6%  0,6% | 4,0% |
| Kaposi sarcoma |  | 2,3% | 2,3% |

Table S3: Anthropometric and biochemical differences from the comparison of KTRs who developed cancer (CA+) and KTRs without cancer diagnosis (CA-).

| **Variables** |  | **CA- (n=753)** | **CA+ (n=177)** | **p value** |
| --- | --- | --- | --- | --- |
| BMI - kg/m^2^ | T1  T12 | 23,2 ± 3,8  24,1 ± 3,9 | 23,9 ± 3,0  24,6 ± 3,1 | **0,018**  0,08 |
| SBP - mmHg | T1  T12 | 130,3 ± 16  129,3 ± 16,3 | 132,3 ± 17  132 ± 17 | 0,06  0,02 |
| DBP- mmHg | T1  T12 | 79,4 ± 10,3  79,4 ± 10,2 | 80,8 ± 10,5  80,4 ± 9,5 | 0,05  0,11 |
| Creatinine - mg/dl | T1  T12 | 1,49 ± 0,65  1,42 ± 0,48 | 1,50 ± 0,50  1,40 ± 0,,38 | 0,39  0,32 |
| Proteinuria - g/24 ore | T1  T12 | 0,31 ± 0,63  0,25 ± 0,44 | 0,25 ± 0,22  0,23 ± 0,35 | 0,14  0,26 |
| Hb - g/dl | T1  T12 | 10,9 ± 1,35  12,7 ± 1,6 | 11,04 ± 1,33  12,9±1,59 | 0,14  0,14 |
| Albumin - g/dl | T1  T12 | 4,1±0,43  4,4±0,47 | 4,1±0,40  4,4±0,36 | 0,40  0,13 |
| Glucose - mg/dl | T1  T12 | 89,8±24  90±24 | 88,5±23,9  87,5±23 | 0,26  0,10 |
| Uric acid- mg/dl | T1  T12 | 5,75 ± 1,6  6,59 ± 2,94 | 5,8 ± 1,5  6,6 ± 1,3 | 0,24  0,39 |
| Total cholesterol - mg/dl | T1  T12 | 210,3 ± 51,4  193,63 ± 43,7 | 216,2 ± 46,4  200,3 ± 39,3 | 0,086  **0,034** |
| HDL cholesterol - mg/dl | T1  T12 | 61,43 ± 20,2  56,5 ± 17,8 | 59,79 ± 16,2  55,92 ± 14,9 | 0,16  0,35 |
| Triglycerides - mg/dl | T1  T12 | 163,5 ± 84,3  146,26 ± 71,3 | 170,96 ± 81,1  153,75 ± 67,1 | 0,14  0,10 |
| 25-OH-vitamin D - ng/ml | T1  T12 | 17,3 ± 8,5  20,2 ± 12,7 | 16,2 ± 6,9  18,1 ± 9,3 | 0,08  **0,033** |

^BMI, body mass index; SBP: systolic blood pressure; DBP: diastolic blood pressure; Hb: hemoglobin.^

**Capsule Sentence Summary**

This monocentric study explores post-kidney transplant cancer. ATG administration, resulting in a significant influence on earlier cancer development, must be a critical and personalized choice. When cancer arises, adjusting immunosuppressive therapy (i.e. mTORi) could be key to achieve better outcomes.


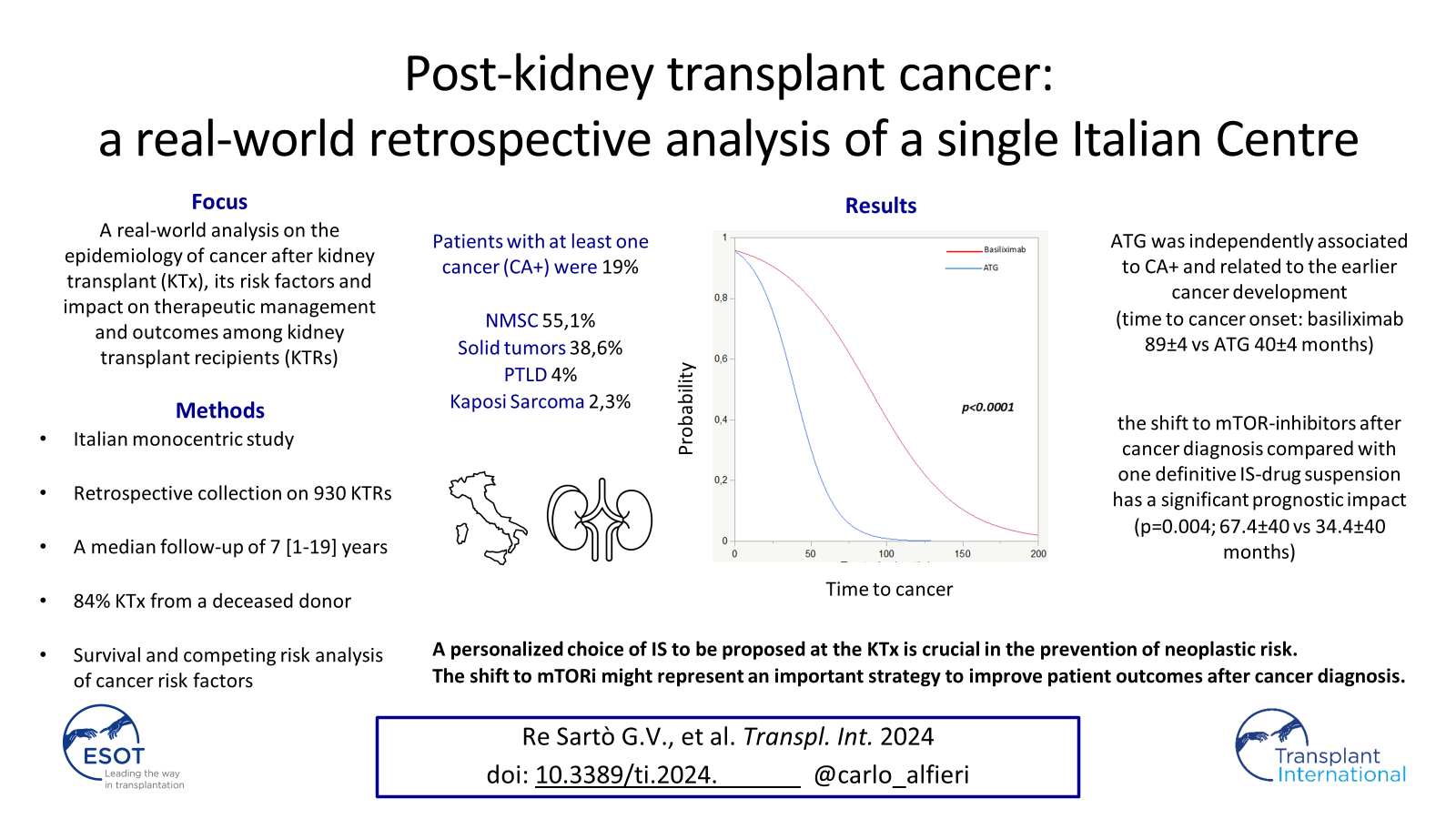

Supplement: Supplementary file 1 [file DataSheet1.docx]
